# Content validation of the SF-36v2® health survey with AL amyloidosis patients

**DOI:** 10.1186/s41687-017-0020-7

**Published:** 2017-12-08

**Authors:** Michelle K. White, Martha S. Bayliss, Spencer D. Guthrie, Kimberly P. Raymond, Avery A. Rizio, Kristen L. McCausland

**Affiliations:** 1 Optum, 1301 Atwood Ave, Suite 311N, Johnston, RI 02919 USA; 2 Prothena Biosciences Inc, 331 Oyster Point Blv, South San Francisco, CA 94080 USA

**Keywords:** SF-36v2, Content validation, Amyloidosis, Quality of life, Qualitative research

## Abstract

**Background:**

This study examined the content validity of the SF-36v2® Health Survey (SF-36v2) in patients with AL amyloidosis using qualitative interviews with physicians and patients. The study included three distinct phases of qualitative research: concept elicitation interviews among physicians, concept elicitation interviews among patients, and cognitive debriefing interviews among patients. The concept elicitation interviews focused on areas of health-related quality of life that are affected by AL amyloidosis and may be affected by treatment, while patient cognitive debriefings aimed to confirm whether the SF-36v2 instructions, recall period, items, and response choices were comprehensive and understandable to AL amyloidosis patients.

**Results:**

Physicians discussed the importance of measuring physical functioning, general health, mental/emotional health, sleep, fatigue, and work impact; though they also reported that they do not routinely use a standard Patient-Reported Outcome (PRO) measure of health-related quality of life. Patients described social, physical, role, and emotional impacts of AL amyloidosis and various treatments. Cognitive debriefing interviews confirmed the relevance of the concepts measured by the SF-36v2 and indicated that patients found the SF-36v2 both easy to understand and complete, that the SF-36v2 instructions and items were comprehensive and understandable without change, and the response choices and recall period were appropriate for use with patients with AL amyloidosis.

**Conclusions:**

The findings support the content validity of the SF-36v2 as an appropriate measure of health-related quality of life in patients with AL amyloidosis.

## Background

Amyloidosis is a disorder in which misfolded proteins lead to the formation of amyloid fibrils, which are deposited in various organs. Amyloid light chain amyloidosis (AL amyloidosis), the most common type of amyloidosis, is characterized by the misfolding of monoclonal light chains, which are secreted by plasma cells [1, 2]. The presence of misfolded light chain proteins is toxic and can cause irreparable tissue damage to organs. Any organ except the brain can be affected by AL amyloidosis [1], with the most commonly involved organs including the heart, liver, gastrointestinal tract, autonomic nervous system, and peripheral nervous system [2]. Deposition of amyloid fibrils in the heart is associated with particularly poor long-term prognosis [3, 4]. Because of variation in the type and number of organ systems involved, symptoms of AL amyloidosis can vary markedly, which can provide additional challenges to both proper diagnosis and treatment. For example, patients with involvement of the autonomic nervous system frequently report diarrhea, while those with kidney involvement may experience edema of the legs [1, 2]. Additional symptoms include macroglossia (enlarged tongue), purpura (bleeding of small blood vessels) on the face or neck, fatigue, weight loss, light-headedness, and carpal tunnel [4–6]. Moreover, the toxic nature of many treatments for AL amyloidosis frequently introduces additional side effects [1].

Given the multitude of symptoms experienced by individuals with AL amyloidosis, a comprehensive understanding of how the disease affects health-related quality of life (HRQoL) is of particular importance. Limited, but consistent, research indicates that patients with AL amyloidosis experience substantial functional burden due to their condition, including significant deficits in both physical and mental aspects of HRQoL, diminished life satisfaction, and greater levels of anxiety and depression [7–14]. Recent reviews have further emphasized the importance of focusing not only on increasing long-term survival, but also on improving patients’ quality of life [2, 12]. To date, the SF-36® Health Survey (SF-36v1 and SF-36v2) is the most frequently used HRQoL measure in studies of patients with AL amyloidosis.

The SF-36v2 is a multipurpose short-form heath survey, and is a widely used generic measure of health status that can target any adult, regardless of disease or treatment group [15, 16]. As such, it is useful for conducting surveys of both general and specific populations, and may be particularly beneficial for use in studies of AL amyloidosis, given the wide heterogeneity among patients in terms of organ involvement and presentation of symptoms. Because of the wide use of this measure, the SF-36v2 can be used to compare the burden of AL amyloidosis experienced by AL amyloidosis patients to that experienced by individuals with other more common diseases; therefore, it can provide context to individuals less familiar with this rare condition. Also, the use of a generic measure may efficiently capture the impact of the diverse set of symptoms and side effects characterized by AL amyloidosis. The SF-36v2 measures eight different health domains using 36 items: physical functioning (10 items), role limitations due to physical problems (four items), bodily pain (two items), general health (five items), vitality (four items), social functioning (two items), role limitations due to emotional problems (three items), and mental health (five items). An additional item assesses change in health status over the past twelve months. Scores derived from the eight health domains can be combined to calculate two additional scores: A physical component summary (PCS) score and a mental component summary (MCS) score, which provide global measures of physical and mental functioning, respectively [16]. A recently developed conceptual model of AL amyloidosis indicated that daily activities, social functioning, and emotional well-being were impacted by the disease [11]. Considering the strong overlap between these areas and the domains assessed by the SF-36v2, this previous research provides preliminary evidence to suggest that the SF-36v2 is a good candidate for assessing HRQoL in individuals with AL amyloidosis.

The purpose of this study was to examine the content validity of the SF-36v2 for use with patients with AL amyloidosis using three qualitative approaches that represent best practices in the field: concept elicitation interviews with physicians, concept elicitation interviews with patients, and cognitive debriefing interviews with patients [17–20]. Although the SF-36 is the most widely used generic measure of HRQoL in clinical trials, with validity established in numerous conditions, it cannot be assumed that the SF-36 is a valid measure in this rare disease population. Thus, this study aimed to investigate which concepts are important to measure to understand the impact of AL amyloidosis on patients’ HRQoL, and to confirm whether the SF-36v2 instructions, recall period, items, and response choices were appropriate, comprehensive, and understandable to patients with AL amyloidosis. Such a validation will also be useful for clinical trials that seek to test the efficacy of a treatment for AL amyloidosis, as the FDA has emphasized the importance of Patient-Reported Outcome (PRO) measures and including the patient perspective in medical product development [17].

## Methods

### Study overview

**Fig. 1:**
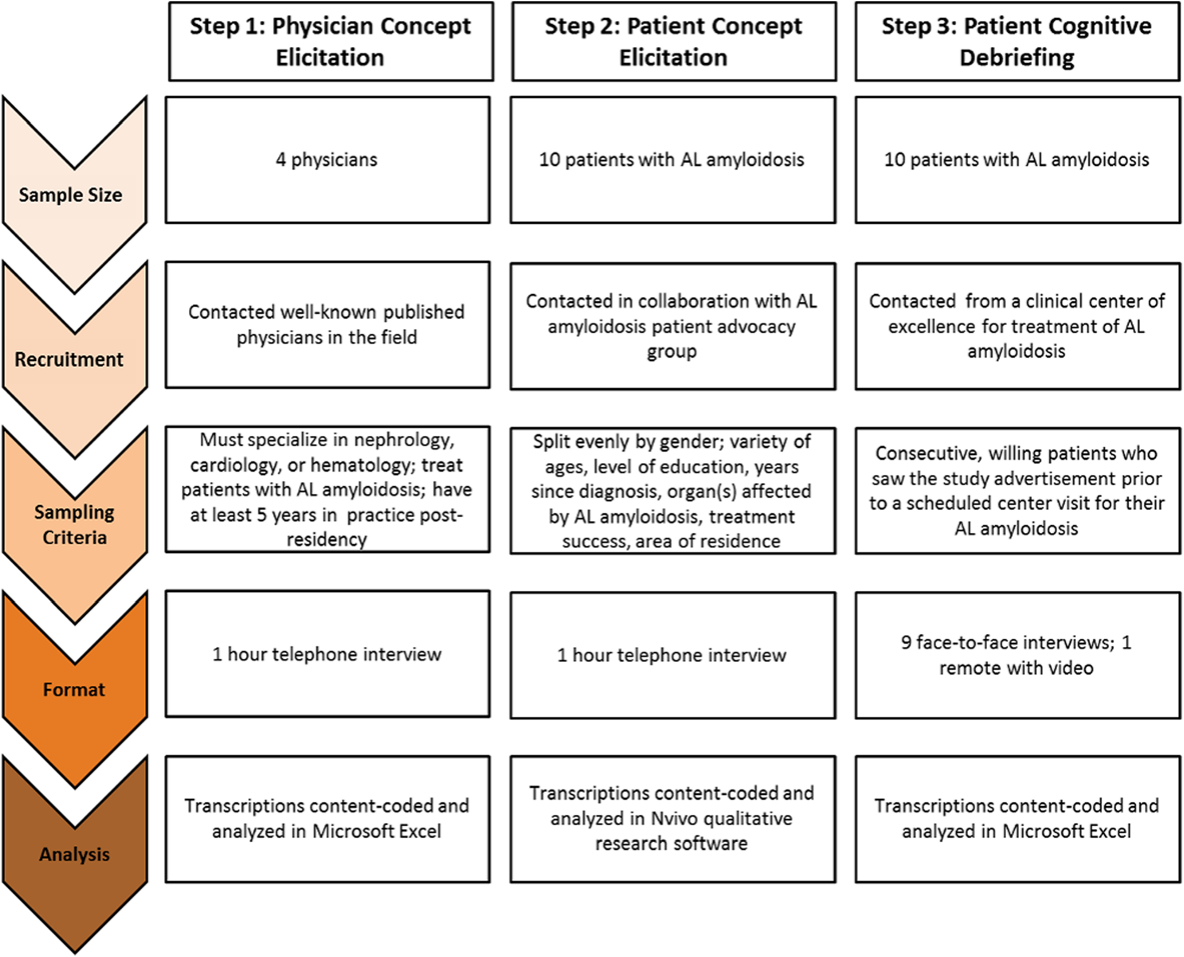
Description of study methodology, divided by interview type

The semi-structured interview guides that were used for each part of the study were informed by a thorough literature review of published reports of HRQoL in patients with AL amyloidosis [8–11, 13, 14]. Inclusion criteria were set so that all patients were at least 18 years of age, self-reported having been diagnosed with AL amyloidosis by a physician, and were able to complete the interview in English. Each interview was audiotaped and transcribed prior to analysis. All patients provided written informed consent, and all study materials were approved by the New England Independent Review Board. An overview of the methods used for each type of interview is presented in Fig. 1, with more specific information documented below.

### Physician concept elicitation

#### Design and procedure

Sampling ensured that the physicians specialized in one of three areas most closely associated with the disease (nephrology, cardiology, hematology). All physicians were required to have treated patients with AL amyloidosis and to have at least five years in practice post-residency. These criteria ensured that the physicians had the experience necessary to appropriately answer the research questions.

Physicians were asked about their experience diagnosing AL amyloidosis; commonly reported symptoms and their impact on patients; and treatment options and challenges. In addition, physicians were asked to consider information related to relevant qualities of a PRO instrument for use with AL amyloidosis patients, including timing (when and how often to use the PRO); recall periods specific to the disease; and which signs, symptoms, or impacts would be most useful to include. Only results relevant to the aims of this study will be reported.

#### Data analysis

Interview transcripts were reviewed and content coded using a grounded theory approach, and were verified by two researchers trained in qualitative data analysis. Any identified discrepancies between coders were reviewed, discussed, and resolved by the research team to help ensure the reliability of the coding. The grounded theory approach allows for themes to emerge from the data rather than imposing a priori hypotheses [21]. For this study, emerging themes generally related to areas of impact related to AL amyloidosis and patients’ quality of life. As new concepts emerged from the data, they were added as new codes.

### Patient concept elicitation

#### Design and procedure

Because of the heterogeneous nature of AL amyloidosis, it is important to consider that patients with different disease characteristics may experience different impacts on HRQoL. As such, sampling procedures ensured that patients were diverse with respect to demographic characteristics such as age and education, and disease characteristics such as years since diagnosis, organs affected by the disease, and treatment success. Patients were recruited through collaboration with an AL amyloidosis patient advocacy group. An email was sent to a random selection of active group members, describing the study and providing information regarding the screening process. Eligible participants were selected based on the inclusion criteria and sampling requirements.

During the concept elicitation, patients were asked to provide information on a variety of topics related to their disease and treatment experiences. Given the goals of the current study, only those findings related to impact of AL amyloidosis on HRQoL will be reported.

#### Data analysis

In addition to the grounded theory approach to coding and analysis described above, an analysis of saturation was conducted with the data from the patient concept elicitation interviews. Saturation is the point at which no new relevant information emerges out of data from sequential interviews, thus providing evidence that enough interviews were completed to fully understand concepts important to patients [21]. To ensure saturation, an iterative approach was used to determine if new concepts were elicited as data analysis progressed and neared completion. The coding process involved first developing a preliminary set of first-level codes by reviewing all 10 interviews. This review facilitated an understanding of the data and helped determine which themes were common across patients. Concepts produced by patients spontaneously were given preference in theme identification. Next, interviews 1–3 were recoded for second-level codes and then coded again using the entire list of new codes. Interviews 4–6 were then coded using the final list of codes, which were followed by the coding of interviews 7–9. Finally, interview 10 was coded to confirm saturation, ensuring that no new concepts emerged. After each set of coding, a review meeting was held amongst coders to ensure rater agreement.

### Patient cognitive debriefing

#### Design and procedure

No formal sampling plan was instituted when recruiting patients for the cognitive debriefing. Instead, patients were recruited for the study consecutively, through collaboration with a clinical center of excellence for treatment of AL amyloidosis. Upon visiting the clinic as part of a two-day series of tests and consultations, patients were given a flyer that described the study and provided contact information for the research team.

Cognitive debriefing interviews with patients followed a standard, in-person, in-depth interview method that has been developed for the SF-36v2 [22]. Each interview began with general questions regarding the impact of AL amyloidosis on the patient. Patients then completed the SF-36v2 (four week recall) in United States (U.S.) English using the think-aloud method [23], which encourages patients to verbalize their thoughts while answering the survey questions. Patients were also asked to describe any aspects of the SF-36v2 they found challenging or confusing. Following spontaneous reports, the interviewer probed areas that appeared to be confusing based on the patient’s facial expression, pauses, or comments. Finally, the interviewer asked a series of questions related to the relevance of the SF-36v2 regarding format, instructions, items, recall period, and each of the response choices, and reviewed a selection of items that patients did not indicate as difficult or challenging. Patients were asked to provide additional feedback or comments on the measure, such as its relevance to AL amyloidosis. The patients who participated in the cognitive debriefing interviews were not the same as those who participated in the concept elicitation interviews, though they were recruited using the same inclusion criteria.

#### Data analysis

Because the purpose of the cognitive debriefing interviews was to confirm the relevance and comprehensibility of the SF-36v2, rather than to explore content themes, a formal saturation coding process and analysis was not required. The narrative data were coded in an Excel database using a series of ratings to systematically summarize patient feedback. The instructions, recall period, and each item and its response choices in the survey were coded to reflect whether a potential problem in understanding was reported spontaneously by the patient, noticed by the interviewer as possibly causing confusion (noted by pauses and facial expressions during the think-aloud process), or reported by a patient after the interviewer probed, specifically asking whether there were any problems understanding that part of the survey. Any potential problem was then also assigned a code as to whether it was a confirmed problem (as opposed to a pause by the patient that did not mean there was a problem in understanding), and whether or not a change could be made to the survey to resolve the problem. This process allowed for the most important elements of the patient reports to be summarized. If a problem was reported, the nature of that problem was also recorded. Prior to coding, a pre-specified threshold of 25% (i.e. three or more patients reporting a consistent problem) of interviews had been set as a guideline for determining whether the reported problem warranted a change, although this was a guideline rather than a rule. Every reported problem was evaluated by the research team to determine whether the requested change was an individual preference or style-related request or a true problem with comprehension.

## Results

### Physician concept elicitation

#### Physician demographics

Characteristics of the four physicians who were interviewed are summarized in Table 1. Three hematologists and one nephrologist, practicing in both the U.S. (*N* = 3) and Europe (*N* = 1) participated. All four physicians worked at academic center hospitals. Physicians were split evenly by gender, and most reported at least ten years of practice post-residency.

**Table 1:** Physician demographic characteristics

Demographic Information	N (%)
Type of Primary Practice	
Hematologist	3 (75)
Nephrologist	1 (25)
Gender	
Male	2 (50)
Female	2 (50)
Region of Practice	
East Coast	2 (50)
Midwest	1 (25)
International (Germany)	1 (25)
Type of Amyloidosis Treated	
AL amyloidosis only	2 (50)
AL amyloidosis & other forms	2 (50)
Years in Practice (post-residency)	
≥ 5 to <10	1 (25)
≥ 10 to <20	3 (75)

#### Assessing HRQoL in patients with AL amyloidosis

Physicians reported that they do ask their patients general questions related to HRQoL, most specifically related to sleep, fatigue, and impacts on work, though they do not routinely use a standardized measure of HRQoL when assessing patients with AL amyloidosis. For example, physicians asked questions such as “*How much energy do you have on a daily basis?*” “*Are you able to work?*” and “*Are you able to do things you enjoy as part of your normal day-to-day life?*”

Physicians noted the importance of assessing physical functioning, general health, mental/emotional health, sleep, fatigue, work impact, and the impact on roles (such as ability to work, participate in family activities, and social functions) in patients with AL amyloidosis. Notably, assessing mental/emotional well-being was regularly reported as equally important to assessing physical functioning, symptoms, and side effects. Physicians were divided, however, in whether problems such as depression and anxiety could be specifically attributed to AL amyloidosis, as they might instead be a common response to learning they have a life-threatening chronic condition. One physician described the emotional problems experienced by patients with AL amyloidosis as similar to patients with cancer, saying:

“So I would classify the emotional symptoms associated with amyloidosis, there’s no physiologic, in fact there is no amyloidosis that involves the brain so the emotional issues that go along with amyloidosis [are]… similar to the lines of patients who have cancers. You know, they’re struck with a very serious life threatening often multi-system disorder…they’re obviously shocked and depressed about that.” *Hematologist, East Coast*

When providing input regarding the recommended frequency of use and appropriate format of an HRQoL assessment for patients with AL amyloidosis, physicians recommended follow-up administration every three months. Once patients achieved a complete hematologic response (i.e. remission), physicians recommend reducing the frequency of assessment to every six months. Physicians also recommended that such an assessment use a recall period of “past month,” because it would most accurately capture potential changes in how patients are feeling, and would not be as strongly affected by day-to-day variability.

### Patient concept elicitation

#### Patient demographics

Characteristics of the concept elicitation patient sample are summarized in Table 2. Patients were split nearly evenly by gender, and represent a range of ages, years since diagnosis, type and number of organs involved, level of education, region of residence (within the U.S.), type of current treatment, and hematologic response to treatment.

**Table 2:** Patient demographic characteristics

	Concept Elicitation	Cognitive Debriefing
Demographic Information	Mean (Range), years	
Age	57.2 (41–76)	63.30 (39–74)
Time since diagnosis	2.3 (0.33–8)	4.18 (0.50–18)
Time from first symptom to diagnosis	2 (0.25–4)	–
	N (%)	
Gender		
Male	6 (60)	5 (50)
Female	4 (40)	5 (50)
Region of U.S. Residence		
South	4 (40)	0 (0)
Midwest	3 (30)	0 (0)
East Coast	2 (20)	10 (100)
West Coast	1 (10)	0 (0)
Education		
Less than Bachelor’s degree	4 (40)	5 (50)
Bachelor’s degree	3 (30)	2 (20)
Post-graduate	3 (30)	3 (30)
Multiple Organ Involvement		
Yes	5 (50)	4 (40)
No	5 (50)	6 (60)
Organs Affected^a^		
Heart	6 (60)	5 (50)
Kidney	5 (50)	6 (60)
Gastrointestinal Tract	3 (30)	0 (0)
Nervous System	2 (20)	1 (10)
Liver	0 (0)	1 (10)
Bladder	0 (0)	1 (10)
Remission Status		
Full Remission	5 (50)	2 (20)
Partial Remission	1 (10)	3 (30)
Not in Remission	4 (40)	4 (40)
Unsure	0 (0)	1 (10)
Current Treatment^a^		
Bortezomib (Velcade)	4 (40)	–
Dexamethasone	3 (30)	–
Doxycycline	2 (20)	–
Lenalidomide (Revlimid)	2 (20)	–
Other	2 (20)	–

^a^Patients were able to select more than one option, so percentages exceed 100

– indicates that data were not collected for this variable

#### Assessing HRQoL in patients with AL amyloidosis

Patients described a variety of ways in which AL amyloidosis affected their HRQoL. Thirteen themes representing areas of impact emerged from the data: social, leisure time, hobbies, mobility, exercise, appetite/food restrictions, family roles, household chores, work, worry, cognition, depression, anger. These areas can be divided into four general areas of impact: social, physical, role, and emotional (see Table 2).

All patients reported impacts related to social functioning. Similarly, patients also frequently discussed the impact AL amyloidosis had on leisure time and hobbies. While some of the effects on social relationships were due specifically to AL amyloidosis, other patients described limitations imposed by treatment regimens and immune-related risks.

Most patients also discussed ways in which AL amyloidosis affected physical aspects of their lives, with nine of the 10 patients specifically invoking limitations on physical mobility, and six of 10 describing how the disease has limited their ability to exercise. In some instances, the limitations on physical activities were quite severe.

Patients consistently discussed how AL amyloidosis had limited their ability to carry out particular roles, including those related to work, family, and household duties. For example, seven of the 10 patients described ways in which AL amyloidosis has affected their work or careers, which included limiting their ability to work, or in some cases causing them to stop working entirely. Others discussed being unable to carry out familial roles or complete household activities, such as chores and housekeeping responsibilities.

The majority of patients (8/10) described at least one way in which AL amyloidosis affected their emotional well-being. Specifically, patients described feeling angry, worried, and depressed as a result of their disease. These negative emotional effects appeared to emanate from a variety of sources, including interacting with doctors and understanding the diagnosis. While patients described anger and frustration related to misdiagnoses, ineffective treatment, and feeling as though physicians seen prior to receiving a diagnosis did not listen to their concerns, these experiences were generally related only to their journey to diagnosis, rather than with respect to their current level of care. In fact, many patients warmly described the ways in which their current physicians care about them.

Overall, the patient concept elicitation interviews demonstrated the varied and oftentimes severe impact that AL amyloidosis has on HRQoL.

### Cognitive debriefing

#### Patient demographics

Characteristics of the cognitive debriefing patient sample are summarized in Table 3. Patients were split evenly by gender, and represent a range of ages, years since diagnosis, type and number of organs involved, level of education, and hematologic response to treatment.

**Table 3:** Patient quotes on impacts of AL amyloidosis on HRQoL

Theme	Subtheme	Number of patients reporting impact (*n* = 10)	Representative patient quotation
Social	Social	10	I used to be very busy with friends and cook and work and run a church. Now, I am mostly home [the] majority of the time because you don’t feel able enough to go out or the medicine that you are on makes it hard for you to go out. *Female, age 53*
	Leisure time	9	So, we had to go home and cancel our life. We had a cruise planned and paid for and birthdays and parties and things like people do. *Female, age 76*
	Hobbies	4	I really, really miss being able to hike, I miss being able to train. I did a lot of training of our animals...and I miss being able to do that a lot because that was very satisfying to me. Those are things that really bother me. *Female, age 49*
Physical	Mobility	9	Bathtubs and showers are the hardest and getting cleaned up, it makes me very short of breath. *Female, age 53*
	Exercise	6	I have worsened to the point where I can’t even walk up a flight of stairs without buckling over. I’m just really struggling for air. *Male, age 41*
	Appetite/Food restrictions	5	I started to lose some of my appetite. Not the least of which is because now I can’t taste… my mouth wasn’t producing much saliva. Everything tastes like cardboard. Disgusting. *Male, age 62*
Roles	Work	7	It’s caused me to have a lot of absences, which I have never had. For a while there it seemed like I was going to the doctor every other day I’m sure that affected my performance at work. Although I tried to be mindful of it, I don’t think you can totally just [block] out all that stuff. *Female, age 63*
	Family roles	9	Even something like getting the screens on the windows. I’ve got to go up a bunch of flights of stairs to do that. I can’t get that done. I feel totally useless in that regard and that’s part of the depression. What am I going to do? Do I put everything on my wife? I can’t do that for God sakes. That has been affected big time. *Male, age 63*
	Household chores	5	Ability to take care of the home is gone. I can’t do anything. *Male, age 63*
Emotions	Anger	3	I just think that people not listening just made me angry. Not that I vented at them, but I felt betrayed. I guess I had always had doctors that listened and I don’t know whether changing from where I live to a bigger city, they just didn’t seem to hear me. *Female, age 60*
	Worry	8	I have so much more concern with, you know, what’s going to happen to [my children]. You know, what happens if I die next year. *Male, age 41*
	Cognition	5	But when I have those spells where I kind of just go away for a while, I think well I’m not able to concentrate, I can’t…how am I going to finish this task? I can’t seem to keep my mind on it. *Female, age 63*
	Depression	5	Not knowing what it is at that point was very scary…It was very depressing at first and very hard to understand because…I didn’t have a clue at first of what it was or what it did or how it would affect my life…It *has* affected it in many different ways. *Female, age 53*

#### Results summary

Cognitive debriefing interviews further confirmed the relevance of the concepts measured by the SF-36v2, as well as the comprehensibility of the SF-36v2 instructions, recall period, items, and response choice sets for patients with AL amyloidosis. Feedback regarding the overall ease of both the measure as a whole and the individual items was consistent, with all patients indicating they found all of the items clear, comprehensible, and easy to respond to. All 10 patients reported that the items presented in the SF-36v2 were relevant. The cognitive debriefing interviews did not reveal any problems with patients’ understanding of items or instructions, and confirmed the appropriateness of the recall period and response options. One recommendation that emerged in the cognitive debriefing patient interviews was for use in a clinical trial, where the expectation is that questions are about their AL amyloidosis, to add a statement prior to the instructions to clarify that patients should respond to the questions on the basis of their overall health rather than just in relation to AL amyloidosis to avoid any confusion. Representative quotations from patients based on key aspects of the SF-36v2 are provided in Table 4.

**Table 4:** Patient quotes from cognitive debriefing interviews

Element of SF-36v2 Survey	Interview Guide Question	Representative Patient Quote
Format and ease	Describe how well you were able to answer the questions overall.	For the most part easy, they weren’t difficult. Trying to determine which shade of something…sometimes you have to think those through. *Male, age 64*
Instructions	Were the instructions easy to understand? Why or why not?	Those were really clear. *Female, age 56*
Item relevance	Which questions were most relevant for describing how you feel and what you are able to do?	I think the daily activity is most relevant. Probably second [most relevant] would be the social. Because I don’t socialize anymore, it is an important area for me. Obviously, [my social life] has been declining as a result of AL amyloidosis. *Male, age 64*
	How do these questions relate to your experience with AL?	Overall they are zeroing in on how I physically am and they are also trying to make sure that my mental health can handle it I guess. It’s apparent. I think those are good issues because, like I said, everybody is different. *Female, age 56*
Ability to detect change over time	Do you think that your answers on this would change as you felt sicker or more well on different medications?	Well, the reality of it is, when the disease is more active or when your symptoms are more active, I think that your answers would tend to be different than when you’re in, and I won’t say I’m in remission, because you don’t have a remission with the disease. But when you’re not being challenged as much by the symptoms of the disease, you would answer it different. *Female, age 59*
Response options	How easy or hard was it for you to answer the questions using the answer choices that are listed?	I would say very easy, and I would say very easy for any person. You know the difference between severe and none. *Male, age 73*
Recall period, 4 weeks	How easy or hard was it for you to think about the past 4 weeks when you were choosing your answers?	… for the past 4 weeks, I can pretty much remember what that’s about. It’s a workable unit. I can think back and say, ‘Oh yeah, in 4 weeks I haven’t…’ *Male, age 67*
Recall period, 12 months	And so the one question that asked about the past year, how difficult was that, to think back for one year?	That’s easy. That’s a good question. I think, over a year, it’s easy. And it’s significant. That’s why they have us doing an annual review over a year, it’s significant. So I have no problem with that. *Male, age 67*
Item content analysis	So now we’re going to go back to any questions that you thought were not clear…Did you mark any that you felt that they weren’t completely clear?	‘I expect my health to get worse.’ I hope not, but I don’t know. Now, guess what? Friday I might know, but right now I don’t know. So they’re kind of a guess, to be honest with you. *Male, age 73*
	So, now we are going to go back to any questions or sections that you thought were not clear, so any place where you might have put a question mark. So, which one was the first that you marked?	'In general, would you say your health is excellent, very good, fair, or poor?' So, it’s subjective. I guess I don’t know if it’s asking me how I feel, like how I feel about my health as opposed to how my health actually is. I guess I could be wrong. So, I could be saying my health is good when it’s actually not. *Female, age 39*

## Discussion

This seminal content validation study of the SF-36v2 for use with patients with AL amyloidosis used a rigorous, three-source approach. It is the first study in the field to explore the usability of a specific measure of HRQoL in patients with this rare disease. The combination of concept elicitation interviews with physicians and patients and cognitive debriefing interviews with patients provide ample evidence that the SF-36v2 appropriately assesses the variety of ways in which AL amyloidosis affects HRQoL. These findings converge with evidence from other sources regarding areas of impact that are relevant to patients with AL amyloidosis (see Table 5).

**Table 5:** Areas of HRQoL impacted by AL amyloidosis mapped onto SF-36v2 domains

Source	Physical Functioning	Role-Physical	Bodily Pain	General Health	Vitality	Social Functioning	Role-Emotional	Mental Health
Literature Review^a^	x	x	x	x	x	x	x	x
Clinician Concept Elicitation	x	x	x	x	x	x		x
Patient Concept Elicitation	x	x	x	x	x	x	x	x
Patient Cognitive Debriefing	x	x	x	x	x	x	x	x
Lin, 2015 Patient Concept Elicitation	x	x	x	x	x	x		x

^a^Includes literature reviewed in the current study and in Lin et al., 2015

Physicians identified many functional limitations commonly seen in their patients that map onto the SF-36v2 domains. This suggests that the measure adequately covers the topics determined to be most relevant by those treating individuals with the disorder. The only topic discussed by physicians that is not evaluated by the SF-36v2 is sleep problems, such as sleep disturbance. However, sleep problems were not frequently reported during patient concept elicitation interviews, leading to the conclusion that its absence on the SF-36v2 will not negatively affect the validity of the instrument in this population. Sleep problems could also be measured using a separate measure. Clinicians and researchers might also consider administering additional PROs designed to capture organ-specific symptoms and impacts. Given differences in both the type and number of organs that can be affected by the disease, pairing the SF-36v2 with an organ-specific measure may provide the best opportunity to fully understand the heterogeneous nature of AL amyloidosis.

Patient concept elicitation interviews provided robust evidence that the SF-36v2 is particularly well suited to capture the varied aspects of HRQoL in this heterogeneous disease. While the SF-36v2 was designed as a measure of generic HRQoL, patients confirmed that the concepts covered by the SF-36v2 are relevant to them and their experiences with AL amyloidosis. Specifically, patients who participated in the concept elicitation interviews described four aspects of HRQoL impairment (social, physical, role, emotional) that are represented by SF-36v2 domains. The majority of *specific* impairments most frequently discussed by patients are also represented on the SF-36v2, which includes items related to limitations in exercise, mobility, work, activities with family, and feelings of depression and anxiety. The current findings also parallel that of previous qualitative work, which has emphasized disease-related impacts on daily activities, social functioning, and emotional well-being [11].

The patient cognitive debriefing interviews provided additional evidence that the SF-36v2 assesses health domains relevant to patients with AL amyloidosis. Patients’ responses to questions regarding both the overall ease of the survey and individual components of the survey such as the instructions and recall period demonstrate that it is easy for patients to understand and complete. Importantly, all patients interviewed described the concepts measured in the SF-36v2 as relevant to them, despite patient differences in factors that influence symptom severity, such as type and number of organs involved, and response to treatment. Patients also reported that the SF-36v2 would accurately capture changes in their HRQoL, which is supported by previous quantitative work showing changes over time in each of the SF-36v2 health domains in patients with AL amyloidosis [10]. Finally, the cognitive debriefing interviews confirmed that the SF-36v2 instructions and items were comprehensive and understandable without change and the response choices and recall period were appropriate for use with patients with AL amyloidosis. Additional research is currently being conducted to explore the quantitative psychometric properties of the SF-36v2 in patients with AL amyloidosis [24].

Similar to most qualitative research in rare diseases, conclusions drawn from the current study are based on a small sample size, particularly with respect to the number of physicians interviewed. In addition, the need to conduct cognitive debriefing interviews in-person resulted in a convenience sample rather than a purposive sample, as study participants lived within driving distance of the interview site and were recruited while visiting their doctors for a routine examination. Despite these limitations, the current study used a sound methodological approach to explore the primary research questions. Future research should include a psychometric validation of the SF-36v2 in a larger sample of patients with AL amyloidosis.

## Conclusions

In conclusion, the results of the current study provided evidence of the content validity of the SF-36v2 as an appropriate measure for assessing the impact of HRQoL in patients with AL amyloidosis. The range and severity of symptoms of this disease, as well as the side effects of treatment, have been shown to greatly impact HRQoL [8, 10, 11, 14], clearly warranting more standardized use of HRQoL measures that are capable of capturing the impacts of such diverse experiences.

## Abbreviations

AL amyloidosis: Amyloid light chain amyloidosis
HRQoL: Health-related quality of life
MCS: Mental component summary
PCS: Physical component summary
PRO: Patient-reported outcome
SF-36v2: SF-36v2® Health Survey
